# Design and Experimental Study of Shape Memory Alloy and Piezoelectric Composite Power Generation Device

**DOI:** 10.3390/mi14071434

**Published:** 2023-07-17

**Authors:** Fengshuang Yang, Yingyu Shi, Jinlong Liu, Zhicong Wang, Xiaochao Tian

**Affiliations:** 1 School of Mechanical and Vehicle Engineering, Changchun University, Changchun 130022, Chinaw18844010813@163.com (Z.W.); 2 China Faw Group Co., Ltd., Changchun 130011, China

**Keywords:** piezoelectric energy capture, piezoelectric film, shape memory alloys, hot engine conversion

## Abstract

In order to solve the problem of ineffective utilization of waste heat generated by energy consumption in industrial production and life, a low-frequency thermal energy conversion type piezoelectric energy trap is proposed, and relevant theoretical analysis and experimental research are conducted. The device utilizes a piezoelectric film (polyvinylidene fluoride) combined with a shape memory alloy and features a simple green structure that can supply energy to microelectronic devices. First, the structural design and working principle of the device are analyzed and the dynamics model is built. Second, COMSOL Multiphysics simulation software (Version 5.6) is used to analyze and calculate the output voltage of shape memory alloy shrinkage, piezoelectric film shape and parameters. Finally, the experimental prototype is machined and fabricated by the fine engraving machine, and the experimental platform is built for relevant performance tests. The experimental results show that when the temperature is 100 °C, the maximum strain of shape memory alloy with 1 mm diameter is 0.148 mm. When the shape of the piezoelectric film is triangular, the length of the bottom edge is equal to the height of the triangle and the thickness ratio is 0.5, the maximum output voltage is 2.12 V. The experimental results verify the feasibility of the designed device and provide new ideas for subsequent research on piezoelectric energy capture.

## 1. Introduction

In recent years, with the rapid development of industrial technology, the traditional battery energy supply can no longer meet the needs of existing instruments, and at the same time, it is harmful to the environment, so the research and development of new energy sources has received wide attention from various countries [1,2,3,4]. Energy as the basis of national economic development and people’s daily lives are closely linked, but the waste heat generated by energy consumption in industrial production is often discharged in the form of free heat dissipation and cannot be effectively utilized on a large scale, which is not in line with the current social development requirements of energy saving and consumption reduction [5,6,7,8,9]. Shape memory alloy has the characteristics of high driving force, high strength and shape memory, which can be combined with piezoelectricity, electromagnetism and electrostatics for composite energy capture, thus forming a secondary energy reuse [10,11,12,13,14,15]. With the advancement of science and technology, piezoelectric materials with high energy density, high response speed and high transduction efficiency are widely used in the field of material energy capture [16,17]. The combination of shape memory alloys and piezoelectric materials for the design and utilization of industrial waste heat capture is a hot topic of research at home and abroad [18].

The main thermoelectric energy capture technologies at this stage are temperature differential energy capture, photoelectric energy capture and pyroelectric energy capture, but they are not applicable to industrial waste heat composite power generation due to external conditions [19,20,21,22,23]. Shape memory alloys and piezoelectric materials are selected to use their own advantages to convert thermal energy to mechanical energy, thus effectively solving the problem of industrial energy saving and consumption reduction. In terms of piezoelectric material selection, piezoelectric films with high service life, high mechanical strength and small size should be selected for subsequent experimental studies. The shape memory alloy and piezoelectric material for capturing energy has received a lot of attention from scholars at home and abroad. For example, Aliae Oudich et al. [24] proposed a memory alloy and piezoelectric ceramic energy trap, and experimental results showed that the maximum output voltage generated by the trap was 63.6 V at a heating temperature of 31 °C with a conversion strain of 0.2% inside the shape memory alloy (SMA) and a thickness ratio of 0.05. Mohamadi R et al. [25] proposed a memory alloy with a piezoelectric cantilever beam-type energy trap and experimental results showed that the shape memory alloy (SMA) achieves an output voltage of 3 V and 13 V at 1% and 2% pre-strain. Qingqing Lu et al. [26] designed a theoretical modeling of a vibrating piezoelectric energy trap, and experimental results showed that the resonant frequency of the device was greatly affected by temperature, and the maximum output voltage generated was 7.738 V at a resonant frequency of 39.4 Hz and an external temperature of 25 °C. Hairong Chen et al. [27] designed a composite material for an energy harvesting study and the experimental results showed that the increase in the pyroelectric coefficient improved the performance of the composite material and increased the power density and efficiency of the piezoelectric ceramic by 25.4%. Because the piezoelectric ceramic plate is composed of PZT and substrate, PZT is liable to break when subjected to external force when the trapper is vibrating, causing the trapper to fail and have low output performance. Piezoelectric films are widely used in the field of piezoelectric energy capture because of their flexibility, long service life and high electromechanical conversion efficiency.

Therefore, this paper proposes an energy trap combining shape memory alloy and piezoelectric film, which can generate the deformation of piezoelectric film through the thermal–mechanical conversion of shape memory alloy and expanding the force by lever structure. The designed energy trap is based on the external temperature variation and can effectively target industrial waste heat for secondary energy use and provide a new idea for composite energy harvesting technology.

## 2. Structural Design and Working Principle

**Figure 1:**
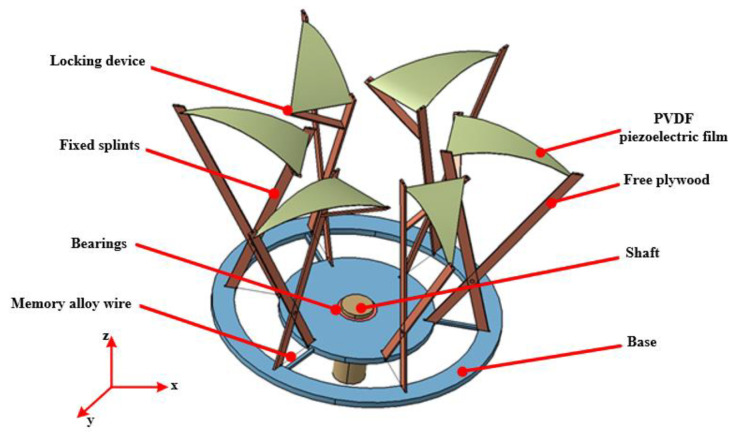
Schematic diagram of the structure of the piezoelectric energy trap.

The three-dimensional structure of the piezoelectric capturer is shown in Figure 1, which mainly consists of shape memory alloy wire (Ni-Ti alloy), PVDF piezoelectric film, shaft, bearing, base, locking device, free clamp and fixed clamp. The dimensions of the fixed and free cleats are 140×20×6mm, the outer ring of the base has a diameter of 180mm, the inner ring has a diameter of 140mm and the shaft has a diameter of 6mm. How the device works: First, a heat gun is used to simulate an external heat source to heat the shape memory alloy, whose internal structure transforms from the martensite to austenite phase and undergoes volume contraction. Second, the memory alloy allows the lower end of the free splint to rotate around the pivot point towards the fixed splint by contraction. Finally, the upper clamp is driven by the lever amplification mechanism to squeeze the piezoelectric film, thus realizing the electromechanical performance conversion.

When the shape memory alloy is not heated, it contains internal austenite and martensite, and when the transformation from austenite to martensite the volume fraction is [28]:

(1) $$\xi_{A\to M}=\frac{\left(1-\xi_{0}\right)\cos\left[a_{M}\left(T-M_{f}\right)+b_{M}\sigma \right]+1+\xi_{0}}{2}$$

the volume fraction during the transformation from martensite to austenite is:

(2) $$\xi_{M\to A}=\frac{\xi_{0}\cos\left[a_{A}\left(T-A_{s}\right)+b_{A}\sigma \right]+\xi_{0}}{2}$$

where A represents austenite, M represents martensite, $M_{f}$ represents the temperature at the end of phase transformation, $A_{s}$ represents the temperature at the beginning of phase transformation. $a_{M}$, $a_{A}$, $b_{M}$, $b_{A}$ are material constants, $a_{A}=\frac{\pi }{A_{f}-A_{s}}$, $a_{M}=\frac{\pi }{M_{s}-M_{f}}$, $b_{M}=-\frac{a_{M}}{C_{M}}$, $b_{A}=-\frac{a_{A}}{C_{A}}$. $C_{M}$ represents the slope of the stress critical value, $C_{A}$ represents the slope of the temperature critical value.

Since the cosine function takes the value range of 0~180 °C, the stress range of the shape memory alloy is:

(3) $$C_{A}\left(T-A_{s}\right)-\frac{\pi }{\left|b_{A}\right|}\leq \sigma \leq C_{A}\left(T-A_{s}\right)$$

(4) $$C_{M}\left(T-M_{f}\right)-\frac{\pi }{\left|b_{M}\right|}\leq \sigma \leq C_{M}\left(T-M_{f}\right)$$

Through equation $\varepsilon =\varepsilon^{e}+\varepsilon^{t}+\varepsilon^{T}$ [29], we obtain the strain of the shape memory alloy with the stress parameters shown in Table 1.

**Figure 2:**
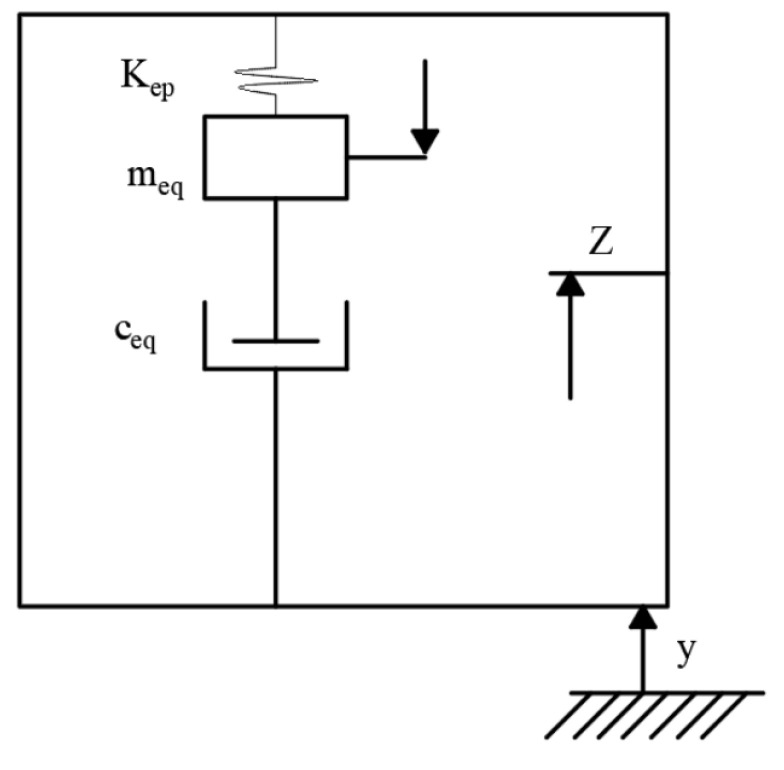
Equivalent model of the piezoelectric module.

Since multiple groups of piezoelectric films are deformed by the extrusion of free plywood, a piezoelectric module is selected to establish a spring-mass-damping model to simulate the vibration deformation of a single-degree-of-freedom system caused by external excitation, and the equivalent model is shown in Figure 2.

The total vibration equation of the piezoelectric module system is:

(5) $$m_{ep}{\ddot{Z}}_{\left(t\right)}+c_{ep}{\dot{Z}}_{\left(t\right)}+k_{ep}Z_{\left(t\right)}=-m_{ep}{\ddot{y}}_{\left(t\right)}$$

where $m_{ep}$ is the total mass, $Z_{\left(t\right)}$ is the mass block displacement, $c_{ep}$ is the damping factor, $K_{ep}$ is the equivalent stiffness and $y_{\left(t\right)}$ is the total piezoelectric film displacement.

Set the external excitation of the free plywood to:

(6) y=asinωt

Bringing Equation (6) into Equation (5) after deriving it twice yields:

(7) $$\sqrt{Z_{\left(t\right)}+2\varepsilon Z_{\left(t\right)}+\omega_{n}^{2}Z_{\left(t\right)}}=\omega \sqrt{a\sin\omega t}$$

Solving Equation (7) to obtain the vibration displacement:

(8) $$\begin{array}{l} z=e^{-\varepsilon t}\left(y_{0}\cos\omega_{\varepsilon }t+\frac{v_{0}+\varepsilon y_{0}}{\omega_{\varepsilon }}\sin\omega_{\varepsilon }t\right)\\ -\bar{a}e^{-\varepsilon t}\left(\sin\phi \cos\omega_{\varepsilon }t+\frac{\varepsilon \sin\phi +\omega \cos\phi }{\omega_{\varepsilon }}\sin\omega_{\varepsilon }t\right)\\ +\bar{a}\sin\left(\omega t+\phi \right) \end{array}$$

where $\phi =\arctan\left(-\frac{2\varepsilon \omega }{\omega^{2}-\omega_{0}^{2}}\right)$, $\bar{a}=\frac{-a\omega^{2}}{\sqrt{\left(\omega^{2}-\omega_{n}^{2}\right)+4\varepsilon^{2}\omega^{2}}}$, ωε=ωn2−ε212 are the system frequencies when damping the system vibration frequency.

Thus, the inertial force generated by the mass block is:

(9) $$F=m_{eq}\left(z+y\right)=-m_{eq}a\omega^{2}\sin\omega t-m_{eq}\bar{a}\omega^{2}\sin\left(\omega t+\phi \right)$$

Under external excitation, the strain at any point of the piezoelectric film is:

(10) $$S_{x}=\frac{z}{R}=\rho z$$

where z is the distance between the amplitude of movement in the *Z*-axis direction and the horizontal, and R is the bending radius of the piezoelectric film.

Then, the cross-sectional bending moment of the piezoelectric film is:

(11) $$M=\int_{a_{1}}\sigma_{1}zdz+\int_{a_{2}}\sigma_{2}zdz$$

The bending moment of the piezoelectric film at any point during external excitation is:

(12) $$M_{x}=F\left(L-x\right)$$

where L is the length of the piezoelectric film.

Then, the expression for the stress in the piezoelectric film is:

(13) $$\sigma_{1}=c_{p}\left(S_{1}+d_{31}E_{3}\right)$$

Substituting Equations (10)–(13) into Equation (11) there are:

(14) $$\begin{array}{l} M=\int_{a_{1}}c_{p}\left(S_{1}+d_{31}E_{3}\right)zdz+\int_{a_{2}}c_{m}S_{m}zdz\\ =\int_{-\delta }^{H_{1}-\delta }bc_{p}\left(-\rho z+d_{31}E_{3}\right)zdz+\int_{-H_{2}-\delta }^{-\delta }bc_{m}\left(-\rho z\right)zdz\\ =\frac{-2b\rho \varphi +3b\psi E_{3}}{6} \end{array}$$

where $\varphi =c_{p}H_{1}\left(H_{1}^{2}-3H_{1}\delta +3\delta^{2}\right)+c_{m}H_{2}\left(H_{2}^{2}-3H_{2}\delta +3\delta^{2}\right)$, $\psi =c_{p}H_{1}\left(H_{1}-2\delta \right)d_{31}$, and b are the widths of the piezoelectric films.

The curvature of the piezoelectric film at any point can be obtained by Equation (15) as:

(15) $$\rho =-\frac{6m_{eq}\omega^{2}S\left(L-x\right)\left(a\omega^{2}\sin\omega t+\bar{a}\omega^{2}\sin\left(\omega t+\phi \right)\right)+3b\psi E_{3}}{2b\varphi }$$

The energy in the system can be obtained from the intrinsic equation of the piezoelectric film, and then its integration gives:

(16) $$u=\int_{V_{1}}\left(\frac{1}{2}c_{p}\rho^{2}Z^{2}-\frac{1}{2}c_{p}d_{31}^{2}E_{3}^{2}+\frac{1}{2}\varepsilon_{33}E_{3}^{2}\right)dV+\int_{V_{2}}\left(\frac{1}{2}c_{m}\rho^{2}Z^{2}\right)dV$$

Substituting Equation (15) into Equation (16) yields the total energy of vibration of the piezoelectric film as:

(17) $$\begin{array}{l} u=\frac{\left[L^{3}-{\left(L-L_{p}\right)}^{3}\right]}{2b\varphi }{\left[-m_{eq}\omega^{2}\left(a\omega^{2}\sin\omega t+\bar{a}\omega^{2}\sin\left(\omega t+\phi \right)\right)\right]}^{2}\\ -\frac{3\psi \left[L^{2}-{\left(L-L_{p}\right)}^{2}\right]}{4\varphi }E_{3}\left[-m_{eq}\omega^{2}\left(a\omega^{2}\sin\omega t+\bar{a}\omega^{2}\sin\left(\omega t+\phi \right)\right)\right]\\ +\left[\frac{3b\psi^{2}L_{p}}{8\varphi }+\frac{1}{2}bL_{p}\left(\varepsilon_{33}-d_{31}^{2}c_{p}\right)H_{1}\right]E_{3}^{2} \end{array}$$

where S is the total area of the piezoelectric film and $L_{P}$ is the length of the piezoelectric layer. Substituting the electric field strength $E_{3}=\frac{V}{H_{1}}$ into Equation (16) and deriving the output voltage yields its open circuit voltage as:

(18) $$v=\frac{q}{c_{pvdf}}=\frac{\frac{3\psi \left[L^{2}-{\left(L-L_{p}\right)}^{2}\right]}{4\varphi H_{1}}m_{eq}\left(a\omega^{2}\sin\omega t+\bar{a}\omega^{2}\sin\left(\omega t+\phi \right)\right)}{\frac{3b\psi^{2}L_{p}}{4\varphi H_{1}^{2}}+\frac{bL_{p}\left(\varepsilon_{33}-c_{p}d_{31}^{2}\right)}{H_{1}}}$$

where a is the bending angle of the piezoelectric film and $H_{1}$ is the thickness of the piezoelectric material.

According to Equation (18), the thickness of the piezoelectric material and the bending angle are positively correlated to the output voltage generated by the capturer, and the bending angle of the piezoelectric film is related to the shrinkage length of the shape memory alloy wire.

## 3. Simulation and Theoretical Analysis

### 3.1. Shape Memory Alloy Shrinkage Analysis

**Figure 3:**
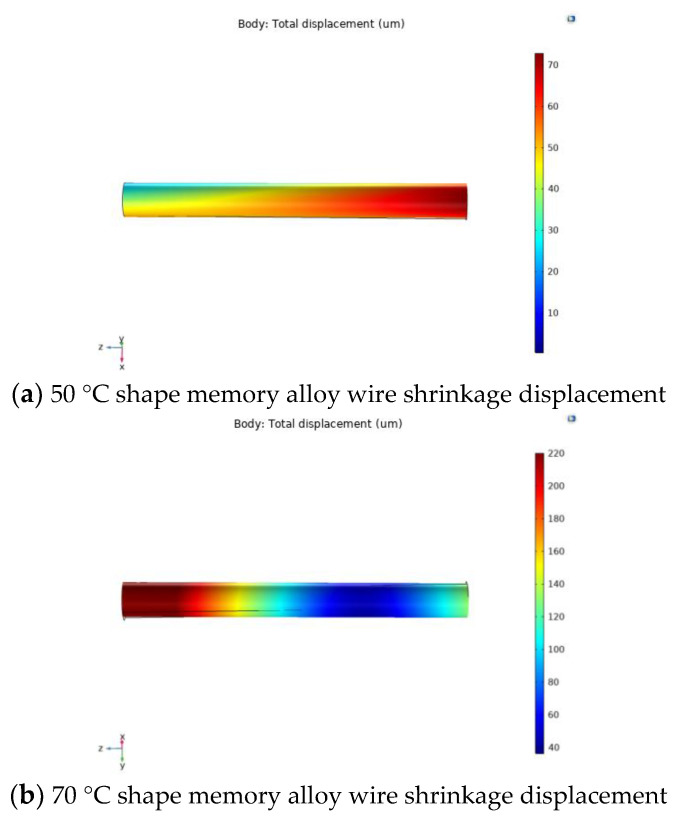

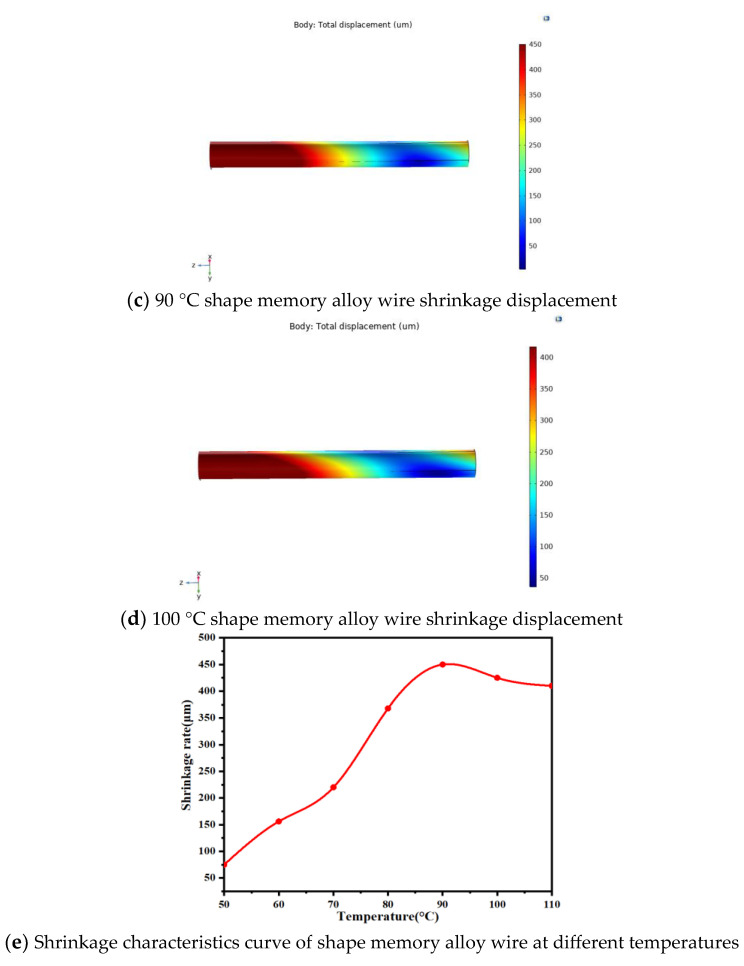
Shrinkage displacement of shape memory alloy at different temperatures.

Since the shape memory alloy is used as the external excitation unit of the piezoelectric energy captor, the output performance produced by the captor is different at different shrinkage rates, so the shape memory alloy wire at different temperatures is simulated using COMSOL Multiphysics software. First, a 3D model with a diameter of 1 mm and a length of 50 mm was imported into COMSOL Multiphysics software. The next material selected for the shape memory alloy is a nickel–titanium alloy and the applied temperature is varied in the physical field. Finally, the steady-state analysis was performed in the study, and the simulation results are shown in Figure 3.

From Figure 3, it can be seen that the shrinkage displacement of the memory alloy wire is increasing with the heat source temperature in a positive correlation. When the temperature of the memory alloy wire is 50 °C, the maximum shrinkage is 0.1394%. When the temperature of the memory alloy wire is 70 °C, the maximum shrinkage rate is 0.4486%. When the temperature of the memory alloy wire is 90 °C, the maximum shrinkage is 0.8354%. When the temperature of the shape memory alloy wire is 100 °C, the maximum shrinkage is 0.829%. Simulation found that the temperature of the memory alloy wire is 90 °C, and the difference between the two shrinkage rates is not too obvious; the above phenomenon may be due to the internal martensite phase all converting to the austenite phase, so that the memory alloy wire does not produce shrinkage.

### 3.2. Piezoelectric Thin Film Voltage Simulation Analysis

**Figure 4:**
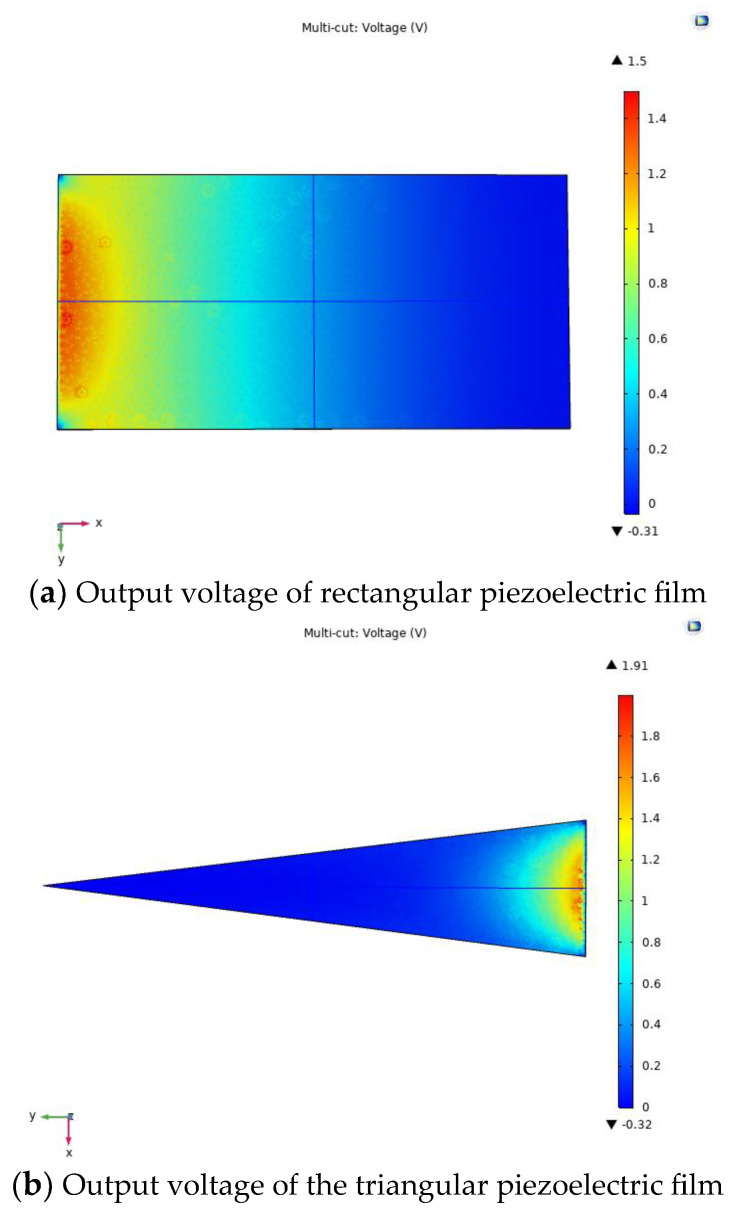

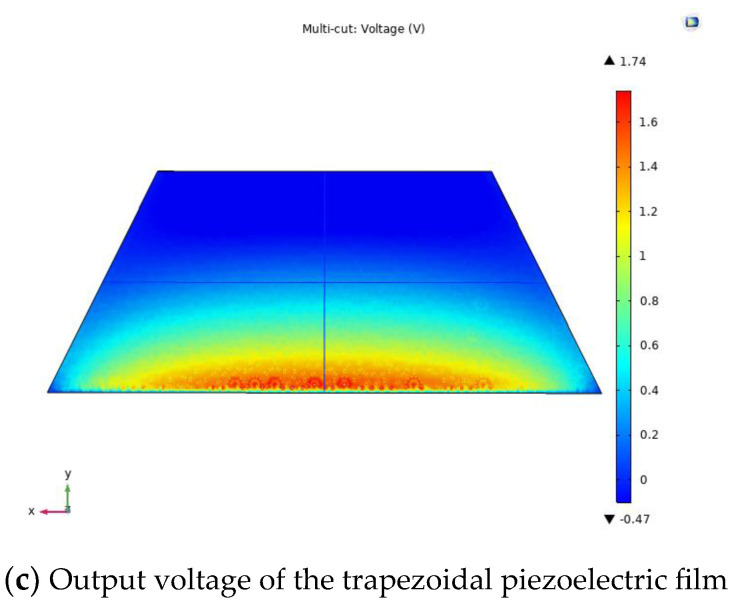
Output voltage in three forms of piezoelectric films.

The piezoelectric film is used as the energy output unit of the trap, and different shapes and sizes of piezoelectric films produce different output voltage performance. Therefore, different shapes and sizes of piezoelectric films were selected for the output voltage simulation analysis, so as to select the film with the best matching performance of the captor. First, the 3D model of the piezoelectric film is imported into COMSOL Multiphysics software, and the film material is selected as PVDF. Second, using the above theoretical equation, the resulting external load is calculated to be 0.5 N, and a fixed constraint is placed on one end of the film in the solid mechanics physical field, and a boundary load of 0.5 N is applied to the upper plane. Finally, meshing is performed and steady-state calculations are performed in the study, and the simulation results for three different shapes and sizes of piezoelectric films are shown in Figure 4. The performance dimensional parameters of the material are shown in Table 2 and Table 3.

As can be seen from Figure 4, the voltage output performance of piezoelectric films of different sizes and shapes varies. The maximum output voltage generated by the triangular piezoelectric film is 1.91 V, the maximum output voltage generated by the trapezoidal piezoelectric film is 1.74 V and the maximum output voltage generated by the rectangular piezoelectric film is 1.5 V. The feasibility of the trap is further verified by simulation, and the triangular piezoelectric film produces the best output performance under the same load and can effectively improve the stress distribution in the piezoelectric film to facilitate electromechanical conversion.

### 3.3. Parametric Analysis of Piezoelectric Films

**Figure 5:**
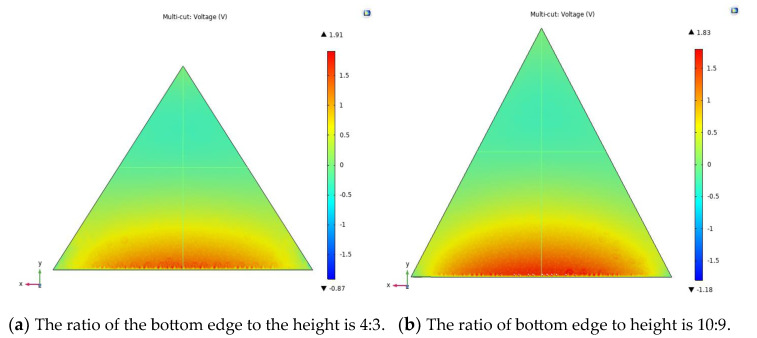

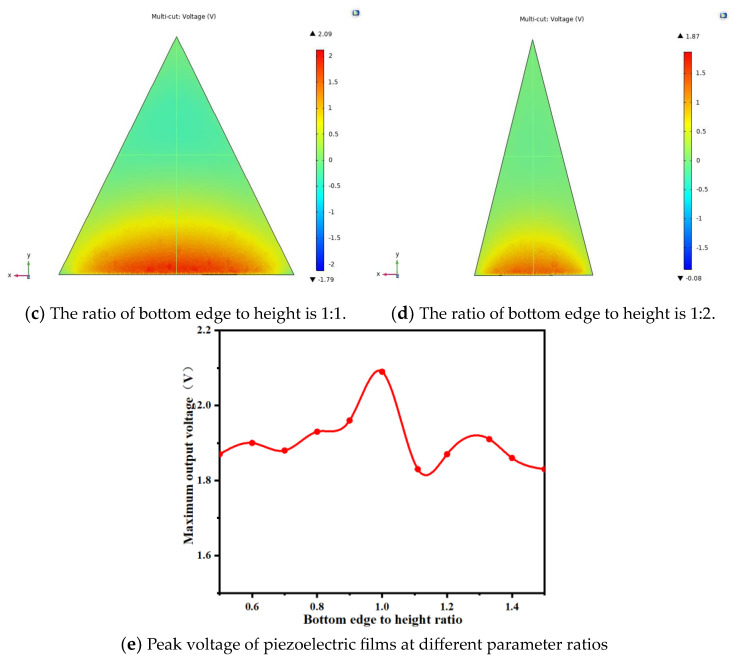
Parametric analysis of triangular piezoelectric films.

As can be seen from Figure 4, the triangular piezoelectric film produces the best output performance. Based on the above study, the ratios were analyzed to further investigate the output performance of piezoelectric films with different size ratios. First, six sizes of piezoelectric films were selected, and the designed piezoelectric film model was imported into COMSOL Multiphysics software, and the piezoelectric material was selected as PVDF. Second, one end of the piezoelectric film is fixedly constrained in the physical field of solid mechanics, and a boundary load of 0.5 N is applied to the upper plane. Finally, the self-using tetrahedral mesh was chosen for the partitioning and research calculations, and the simulation results for the six sizes of films are shown in Figure 5.

As can be seen from Figure 5e, different ratios of piezoelectric films produce different output voltages. When the bottom edge of the piezoelectric film is equal to the height, the resulting output voltage is a maximum of 2.09 V. The feasibility of the capturer is further verified through simulations to provide a theoretical basis for subsequent experimental tests.

## 4. Experimental Testing

### 4.1. Shrinkage Performance Testing of Shape Memory Alloys

**Figure 6:**
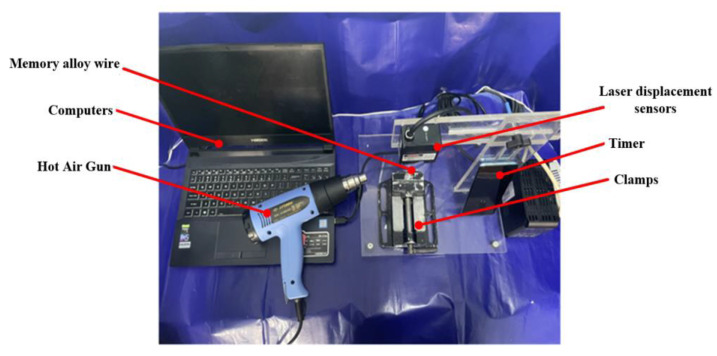
Shrinkage performance experimental test platform.

**Figure 7:**
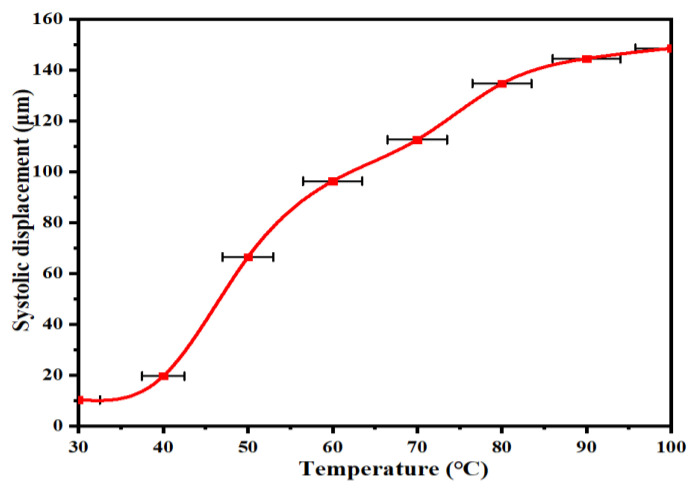
Shrinkage displacement of shape memory alloys at different temperatures.

In order to verify that the shape memory alloy has a certain shrinkage rate, the experimental test platform is built, as shown in Figure 6. It mainly includes: shape memory alloy wire, laser displacement sensor, fixture, hot air gun, timer and computer. First, the shape memory alloy wire with a diameter of 1 mm and a length of 50 mm is selected and a heat gun is used as a heat source to bring it to the corresponding temperature. Next, the shrinkage displacement of the memory alloy wire is measured using a laser displacement sensor and the time of shrinkage is recorded by a stopwatch. Finally, the measured data were imported into the computer for data processing. Since there may be inhomogeneity in heat transfer from the heat gun to the shape memory alloy wire, the error estimation of the heat transfer process was performed using equation $\Phi =\alpha (T_{w}-T_{f})A$ based on the convective heat transfer mechanism, and the experimental results are shown in Figure 7.

As can be seen from Figure 7, the shrinkage of the shape memory alloy tends to increase as the temperature of the heat source increases. When the heating temperature is 100 °C, the maximum strain produced by the memory alloy wire is 0.148 mm. Experimental testing of shrinkage performance is conducted to verify the feasibility of memory alloy as an excitation source.

### 4.2. Test of Shape Memory Alloy Wire Diameter on Film Output Voltage

**Figure 8:**
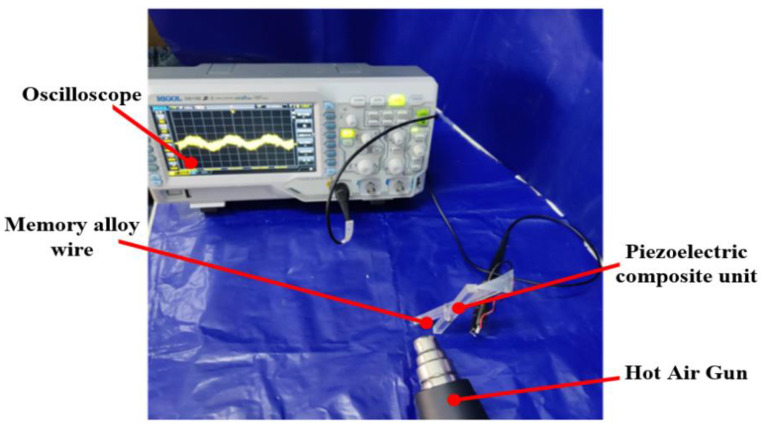
Experimental test setup of the composite unit.

Because the shape memory alloy acts as the excitation source of the capturer and acts on the piezoelectric film through the lever amplification mechanism, whether different diameters of the shape memory alloy wire will have an effect on the output performance of the capturer must be deduced. An independent composite unit is selected for experimental testing using a heat gun with an oscilloscope, and the experimental setup is shown in Figure 8.

**Figure 9:**
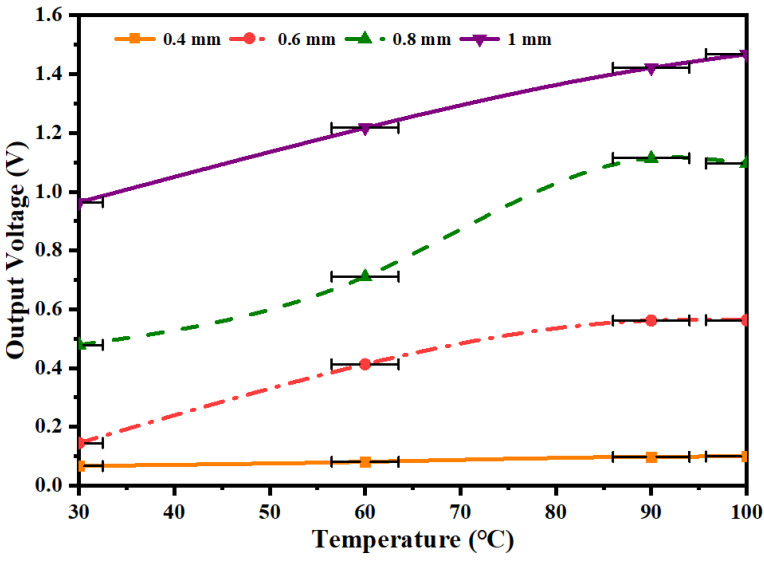
Output voltage characteristic curves of films with different shape memory alloy wire diameters.

In order to investigate the effect of different alloy wire diameters on the output voltage of the capturer, a heat gun was used to adjust the temperature to 30~100 °C, and the shape memory alloy wire diameters of 0.4 mm, 0.6 mm, 0.8 mm and 1 mm were selected for experimental testing, and the experimental results are shown in Figure 9.

As can be seen from Figure 9, the output voltage of the film tends to increase as the temperature of the heat source rises. When the heat source temperature is 100 °C, the shape memory alloy wire diameter is 1 mm, and the output voltage of the film reaches a maximum of 1.468 V. The reason for this phenomenon may be due to the larger driving force generated by the 1 mm diameter shape memory alloy wire, which is amplified by the lever mechanism, resulting in a significant increase in the amplitude of the output voltage generated by the film.

### 4.3. Test of Different Piezoelectric Film Shapes on the Output Voltage of the Trap

**Figure 10:**
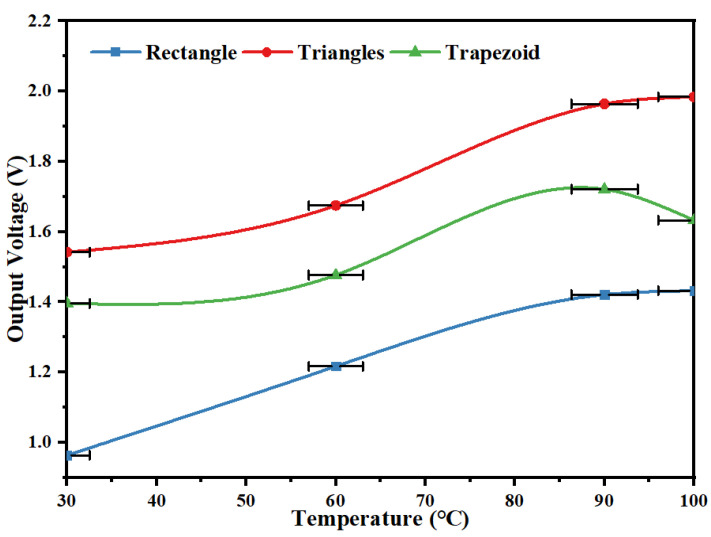
Output voltage characteristic curves of piezoelectric films at different temperatures.

To investigate the effect of the shape of the piezoelectric film on the output voltage of the trap, the trapezoidal, rectangular and triangular piezoelectric films were selected, and the diameter of the shape memory alloy wire was 1 mm. The output voltages of three different shapes of piezoelectric films were tested with the capturer at 30~100 °C. The experimental test results are shown in Figure 10.

As can be seen from Figure 10, all three film shapes show an increasing trend in output voltage with increasing temperature. When the temperature reaches 100 °C, the maximum output voltage produced by the triangular piezoelectric film is 1.994 V, the maximum output voltage produced by the trapezoidal piezoelectric film is 1.721 V, and the maximum output voltage produced by the rectangular piezoelectric film is 1.432 V.

### 4.4. Effect of Different Film Parameters on the Output Performance of Energy Traps

**Figure 11:**
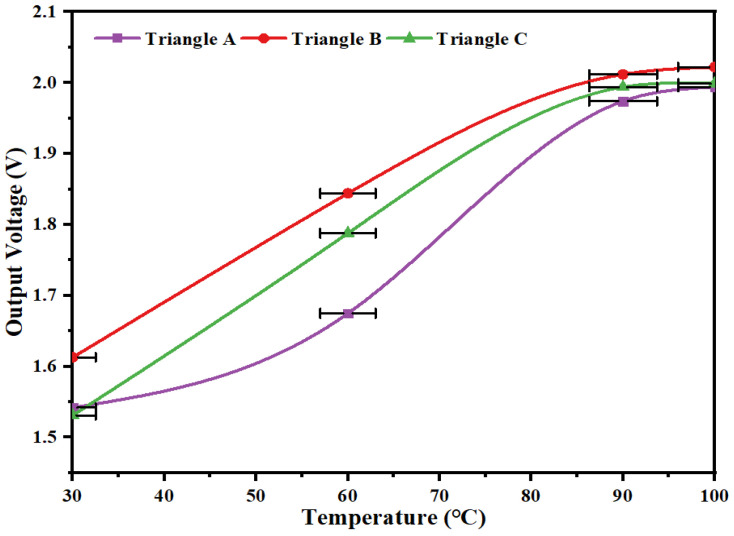
Output voltage characteristic curves of piezoelectric films with different size parameters.

In order to investigate the optimal performance of the triangular piezoelectric film, the parameters of its bottom edge and height dimensions were varied and the output performance of the capturer at 30~100 °C was investigated using the experimental setup detailed above. The piezoelectric film size parameters are shown in Table 4, and the experimental results are shown in Figure 11.

From Figure 11, it can be seen that the output performance of the triangular piezoelectric film is optimal when the triangular bottom edge and height dimensions are equal. When the temperature reaches 100 °C, the maximum output voltage is 2.02 V. The rationality of the designed model is further verified by comparing it with the simulation results.

### 4.5. Effect of Different Piezoelectric Film Thickness on Output Performance

**Figure 12:**
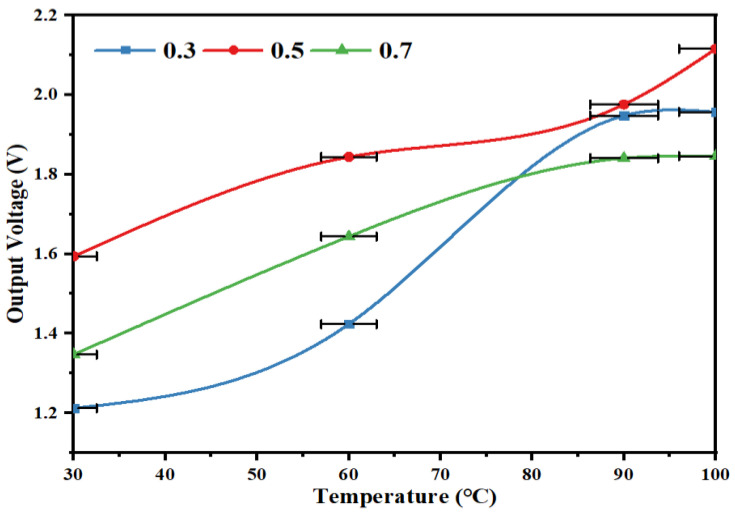
Output voltage characteristic curves of piezoelectric films at different thicknesses.

In order to investigate whether different thicknesses of piezoelectric films have an effect on their output performance, piezoelectric films with a heat source temperature of 30~100 °C, and substrate-PVDF thickness ratios of 0.3, 0.5 and 0.7 were selected for performance testing, and the experimental results are shown in Figure 12.

As can be seen from Figure 12, the output voltage of the piezoelectric film tends to increase as the temperature increases, but does not keep increasing with the increase of the substrate thickness ratio. When the thickness ratio of the piezoelectric film is 0.5, the output voltage is 2.12 V maximum.

### 4.6. LED Lighting Experiment Test

**Figure 13:**
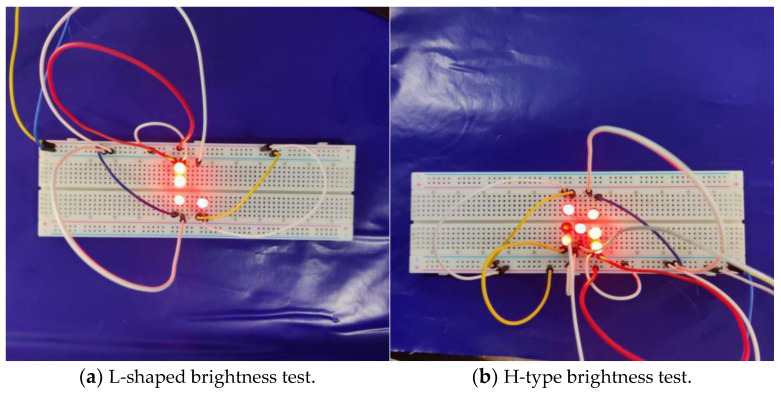
LED light test results.

In order to verify that the designed energy capturing apparatus has certain energy capturing effect, the experimental analysis is carried out using LED lights. First, the LED lights will be divided into two groups, the first group consists of four LED lights in series and form the English letter “L” type. The second group consists of 7 LEDs connected in parallel and form the letter “H”. Second, the energy captor was connected directly to the LED lamp through the rectifier, the intermittent heating of the shape memory alloy wire was started using a heat gun, and the experimental results are shown in Figure 13.

As can be seen in Figure 13, the LEDs in the experimental setup were able to be lit, thus verifying the feasibility of the energy capturer. By using a hot air gun to shape the memory alloy wire heating 69 min after stopping the heating, the LED lamp consumes electrical energy in the capacitor 7 min after the electrical energy in the capacitor is consumed and the LED lamp is off; its electromechanical conversion efficiency is about 10.14%.

## 5. Conclusions

This paper proposes a thermos-mechanical conversion type captive energy device, which mainly consists of piezoelectric film, shape memory alloy, free clamping plate, fixed clamping plate, locking device and base. The designed energy trap can effectively target industrial waste heat for secondary energy use. Second, COMSOL Multiphysics simulation software is used to analyze and calculate the output voltage of the shape memory alloy shrinkage rate, piezoelectric film shape and parameters. Finally, the experimental prototype is machined and fabricated using a precision engraving machine to build an experimental platform for relevant performance tests. The experimental results show that the optimal power generation is 2.12 V when the heat source temperature is 100 °C, the shape memory alloy wire diameter is 1 mm, the shape of the piezoelectric film is a triangle, the length of the bottom edge is equal to the height of the triangle, and the thickness ratio is 0.5. This experiment verifies the feasibility of the thermo-mechanical conversion-type energy capturer, which can be placed at industrial waste heat with power generation and energy saving, and thus provides new ideas for the future piezoelectric energy capturing field.

## Figures and Tables

**Table 1:** Stress parameters of shape memory alloys.

Material Parameters	C1	C2	C3	C4	C5	C6
Value (Mpa)	520	600	300	200	0.07	0

**Table 2:** PVDF piezoelectric film material parameters.

Materials	Modulus of Elasticity (GPa)	Density (kg/m^3^)	Young’s Modulus (GPa)	Poisson’s Ratio
PVDF	2.5	1780	1.12	0.3

**Table 3:** PVDF piezoelectric film dimensional parameters.

Shape	Length/Bottom (mm)	Width/Height (mm)	Thickness (mm)
Triangles	20	80	0.2
Rectangle	40	20	0.2
Trapezoid	Upper base 30, lower base 50	20	0.2

**Table 4:** Dimensional parameters of piezoelectric films.

	Bottom Edge (mm)	Height (mm)	Thickness (mm)
Triangle A	20	80	0.2
Triangle B	40	40	0.2
Triangle C	53	30.2	0.2

## Data Availability

The data that support the findings of this study are available from the corresponding author upon reasonable request.
